# The impact of neoadjuvant and adjuvant immunotherapy on the survival of pancreatic cancer patients: a retrospective analysis

**DOI:** 10.1186/s12885-020-07016-8

**Published:** 2020-06-09

**Authors:** Saber Amin, Michael Baine, Jane Meza, Chi Lin

**Affiliations:** 1 Department of Radiation Oncology, University of Nebraska Medical Center, 986861 Nebraska Medical Center, Omaha, NE 68198-6861 USA; 2 Department of Biostatistics, College of Public Health, University of Nebraska Medical Center, Omaha, USA

**Keywords:** Neoadjuvant therapy, Adjuvant therapy, Immunotherapy, Overall survival, Pancreatic adenocarcinoma

## Abstract

**Background:**

Immunotherapy has become an essential part of cancer treatment after showing great efficacy in various malignancies. However, its effectiveness in pancreatic ductal adenocarcinoma (PDAC), especially in resectable pancreatic cancer, has not been studied. The primary objective of this study is to compare the OS impact of immunotherapy between PDAC patients who receive neoadjuvant immunotherapy and patients who receive adjuvant immunotherapy. The secondary objective is to investigate the impact of neoadjuvant and adjuvant immunotherapy in combination with chemotherapy and chemoradiation by performing subset analyses of these two groups.

**Methods:**

Patients diagnosed with PDAC between 2004 and 2016 were identified from the National Cancer Database (NCDB). Multivariable Cox proportional hazard analysis was performed to examine the effect of neoadjuvant and adjuvant immunotherapy in combination with chemotherapy and chemoradiation on the OS of the patients. The multivariable analysis was adjusted for essential factors such as the age at diagnosis, sex, race, education, income, place of living insurance status, hospital type, comorbidity score, and year of diagnosis.

**Results:**

Overall, 526 patients received immunotherapy. Among whom, 408/526 (77.57%) received neoadjuvant immunotherapy, and the remaining 118/526 (22.43%) received adjuvant immunotherapy. There was no significant difference in OS between neoadjuvant and adjuvant immunotherapy (HR: 1.06, CI: 0.79–1.41; *p* < 0.714) in the multivariable analysis. In the univariate neoadjuvant treatment subset analysis, immunotherapy was associated with significantly improved OS compared to no immunotherapy (HR: 0.88, CI: 0.78–0.98; *p* < 0.026). This benefit disappeared in the multivariable analysis. However, after patients were stratified by educational level, the multivariable Cox regression analysis revealed that neoadjuvant immunotherapy was associated with significantly improved OS (HR: 0.86, CI: 0.74–0.99; *p* < 0.04) compared to no immunotherapy only in patients with high-level of education, but not in patients with low-level of education.

**Conclusion:**

In this study, no difference in the OS between patients who received neoadjuvant immunotherapy and patients who received adjuvant immunotherapy was noticed. Future studies comparing neoadjuvant adjuvant immunotherapy combined with chemotherapy, radiation therapy, and chemoradiation are needed.

## Background

The majority of pancreatic ductal adenocarcinoma (PDAC) patients are diagnosed with unresectable PDAC, while less than 20% are diagnosed with resectable cancer [1, 2]. The current standard-of-care treatment for resectable PDAC is upfront surgery followed by adjuvant single or combined chemotherapy [3]. The median overall survival (OS) after surgery is between 15 and 24 months with a five-year survival rate of 20% with some recent data showing a median OS of up to 54 months [4–7]. Up to 80% of patients who undergo surgery experience recurrence, owing significantly to micrometastases, which occur early in the disease, or microscopic residual disease in the tumor bed [1, 2]. These difficulties have brought adjuvant therapy to the forefront of PDAC treatment. In a recent phase III randomized trials, the combination of adjuvant gemcitabine and capecitabine was associated with better median OS (28·0 months, 95% CI 23·5–31·5 vs. 25.5 months, CI: 22.7–27.9) compared to the gemcitabine alone group (hazard ratio 0·82, CI 0·68–0·98; *p* = 0·032). The combination of gemcitabine and capecitabine should be the treatment of choice in the adjuvant setting after the surgical resection of adenocarcinoma [8]. Nonetheless, despite improvement in surgical techniques, radiation therapy (RT), and chemotherapeutic options, only a modest improvement in the OS has been noticed [9]. Due to the lack of current standard-of-care treatments, novel treatment strategies such as the use of immunotherapeutics are desperately needed.

Immunotherapy has worked well in many solid cancers, but its use in PDAC is not clear [10, 11]. The use of immunotherapy to date has been mainly in a metastatic setting. However, new evidence indicates that immunotherapy could be useful in patients with localized disease who have a high risk of micrometastases [12–17]. Chemotherapy and RT increase tumor-specific T cell infiltration, decrease T regulatory cells, and suppress Myeloid-derived suppressor cells (MDSCs), and can have synergistic interaction with immunotherapy [15, 18, 19]. Immunotherapy was associated with tumor regression and improved OS in preclinical studies of PDAC when used in combination with other treatments [20, 21]. To achieve the optimal OS effect of the use of immunotherapy with chemotherapy and chemoradiation in PDAC, the sequence of the treatment is critical. The sequence of treatment even becomes more significant in resectable PS due to the potential interactions of systemic therapy with surgery. Due to the higher rate of recurrence after surgery, the early implementation of systemic therapy is needed [22].

Neoadjuvant treatment (NAT) strategies have emerged and been employed as an attractive option for resectable and potentially resectable PDAC [23, 24]. Neoadjuvant treatment can also turn those initially borderline resectable or even some unresectable disease into resectable [23, 24]. This strategy provides an opportunity for an early start of systemic therapy in contrast to upfront surgery, where more than half of the patients may not receive adjuvant therapy due to postoperative complications and declining performance status [25–27]. Recent clinical trials and systematic reviews have reported the survival benefit of NAT [28–31]. However, the effectiveness of NAT in resectable PDAC remains unclear as there are still many questions to be addressed before NAT becomes a standard of care [32].

The neoadjuvant and adjuvant use of immunotherapy both could be justified. Neoadjuvant immunotherapy with chemotherapy or chemoradiation could shrink the tumor, downstage nodal disease, and increase the chance of margin negative resection as reported for neoadjuvant systemic therapy [33, 34]. It may also work with chemotherapy or chemoradiation to mitigate the risk of micrometastases [35, 36]. Contrary, adjuvant immunotherapy may be effective when the bulk of the tumor is removed, and there is a minimal residual disease, which T cells can target and eliminate. In addition, the timing of adjuvant immunotherapy needs to be appropriately chosen as surgery is associated with transient immunosuppression [37, 38]. The use of immunotherapy in neoadjuvant or adjuvant setting combined with chemoradiation in PDAC has been limited. Some clinical trials studying the efficacy of immunotherapy in resectable PDAC combined with chemoradiation therapy have shown positive response and measurable activity [39–42]. However, extensive studies of neoadjuvant and adjuvant immunotherapy in resectable PDAC are lacking. The objective of this study is to investigate the impact of neoadjuvant and adjuvant immunotherapy in combination with chemotherapy and chemoradiation on the OS of resectable PDAC patients using the National Cancer Database (NCDB).

## Methods

### Data source

The data for this study were extracted from a de-identified file of the National Cancer Database (NCDB). The NCDB is a joint program of the Commission on Cancer of the American College of Surgeons and the American Cancer Society. It captures 70% or more of newly diagnosed malignancies in the United States annually. This study was exempt from the Institutional Review Board (IRB) because the de-identified data were used.

### Study population

The study included patients age 18 or older who were diagnosed with PADC between 2004 and 2016 and received definitive surgery of the tumor. The ICD-O-3 histology codes of 8000, 8010, 8020–8022, 8140, 8141, 8211, 8230, 8500, 8521, 8050, 8260, 8441, 8450, 8453, 8470–8473, 8480, 8481, 8503,8250,8440, 8560 were used to identify PDAC. The surgical site-specific code was used to identify patients with definitive surgery of the pancreas. Patients with missing information related to RT, chemotherapy, immunotherapy, and sequence of these treatments with each other and surgery were excluded. Patients with the M1 stage and those with unknown or missing information about other covariates in the adjusted multivariable analysis were also excluded. The analysis of the sequence of immunotherapy with RT alone was not performed due to the small sample size. The variable of days from diagnosis to the start of the treatment was used to identify neoadjuvant and adjuvant immunotherapy, chemotherapy, and chemoradiation. If chemotherapy, RT, and immunotherapy were delivered more than eight months before or after surgery, those patients were excluded. If immunotherapy was received more than six months before or after chemotherapy or RT, those patients were also excluded. The primary outcome of the current study was the OS of the patients, which was calculated from the date of diagnosis to the date of death. Patients who were alive or lost to follow up were censored. The subset analysis of the neoadjuvant group only included patients who received only neoadjuvant chemotherapy, immunotherapy, RT, and chemoradiation. If any of the treatment was not neoadjuvant, they were excluded from the subset analysis. Patients with no treatment were also excluded. The subset analysis of adjuvant treatment comparison included patients who only received adjuvant chemotherapy, immunotherapy, RT, and chemoradiation. If any of the treatment was not adjuvant, those patients were excluded for adjuvant subset analysis. Patients with no treatment were also excluded from this subset analysis.

### Explanatory variables

The main predictors of OS in this study were immunotherapy combined with chemotherapy, and immunotherapy combined with chemoradiation. The age at diagnosis, gender, race, urban and rural living status, income, education, treatment facility type, comorbidity score, insurance status, year of diagnosis, and receipt of chemotherapy, RT, and immunotherapy were other explanatory variables used in the multivariable analysis.

### Statistical analyses

Descriptive statistics for categorical and continuous variables are reported. A Chi-square test was used to report the association of the explanatory variables with the treatment sequence of immunotherapy with chemotherapy and chemoradiation therapy. The difference in the median OS between the different treatment sequences was reported using the Kaplan-Meier (KM) curves based on the log-ranks test. The Cox regression analysis was used to study the effect of different variables on OS. The estimated hazard ratio (HR) with its associated 95% confidence intervals (CI) was reported. A p-value of 0.05 was considered significant. The analysis was conducted using the SAS 9.4 software (SAS Enterprise, Cary, NC).

## Results

### Neoadjuvant immunotherapy vs. adjuvant immunotherapy

Among 526 patients who received immunotherapy, 408/526 (77.57%) received neoadjuvant immunotherapy, and the remaining 118/526 (22.43%) received adjuvant immunotherapy. The median age at diagnosis among patients who received immunotherapy was 62 with a range of (29–88) years. The median age at diagnosis of patients who received neoadjuvant immunotherapy was 62.0 (34–88) years, while it was 62.5 (29–86) years in patients who received adjuvant immunotherapy. A majority of the patients were White, living in the urban areas, had a high school degree, had income > = $35,000, had insurance, were treated in academic hospitals, and had a Charlson/Deyo Score of zero. There was no association between the baseline characteristics of the patients and receiving neoadjuvant or adjuvant immunotherapy except the year of diagnosis. Among patients who received neoadjuvant immunotherapy, 41.67% were diagnosed after 2011, while among patients who received adjuvant immunotherapy, 66.10% were diagnosed after 2011. Among those diagnosed after 2011, 68.55% received neoadjuvant immunotherapy compared to 31.45% who received adjuvant immunotherapy, while among those who were diagnosed before 2011, 85.61% received neoadjuvant immunotherapy compared to 14.39% who received adjuvant immunotherapy. The baseline characteristics of the study population are shown in Table 1. We did not report the baseline characteristics of the neoadjuvant and adjuvant subset analyses due to insignificant results of these subsets.

**Table 1:** Baseline characteristics of neoadjuvant vs. adjuvant immunotherapy

Variable		Neoadjuvant immunotherapy 408 (77.57%)	Adjuvant immunotherapy 118 (22.43%)	Total 526	P
Age at diagnosis: median (range)		62 (34 -88)	62.5 (29-86)	62.00 (29–88)	0.541
Sex	Male	238 (58.33)	62 (52.54)	300 (57.03)	
	Female	170 (41.67)	56 (47.46)	226 (42.97)	0.263
Race	White	359 (90.43)	113 (96.58)	472 (91.83)	0.049
	Black	21 (5.29)	4 (3.42)	25 (4.86)	
	Other	17 (4.28)	0 (0.00)	17 (3.31)	
	Unknown	11	1	12	
Education	> = 13% HG	112 (27.72)	29 (24.58)	141 (27.01)	0.498
	< 13%	292 (72.28)	89 (75.42)	381 (72.99)	
	Unknown	4	0	4	
Income	> = $35,000	292 (72.28)	89 (75.42)	381 (72.99)	
	< 35,000	112 (27.72)	29 (24.58)	141 (27.01)	0.498
	Unknown	4	0	4	
Place of Living	Urban	384 (98.71)	115 (99.14)	499 (98.81)	
	Rural	5 (2.29)	1 (0.86)	6 (1.19)	0.712
	Unknown	19	2	21	
Hospital Type	Academic	318 (78.91)	95 (82.61)	413 (79.73)	
	Community	85 (21.09)	20 (17.29)	109 (20.27)	0.384
	Unknown	5	3	8	
Insurance Status	Insured	398 (98.51)	118 (100.00)	516 (98.85)	
	Not insured	6 (1.49)	0 (0.00)	6 (1.15)	0.183
	Unknown	4	0	4	
Charlson/Deyo Score	0	303 (74.26)	88 (74.58)	391 (74.33)	
	1	89 (21.81)	25 (21.19)	114 (21.67)	
	> = 2	16 (3.92)	5 (4.24)	21 (3.99)	0.980
Year of Diagnosis	2004–2010	238 (58.33)	40 (33.90)	278 (52.85)	0.0001
	2011–2016	170 (41.67)	78 (66.10)	248 (47.15)	

**Fig. 1:**
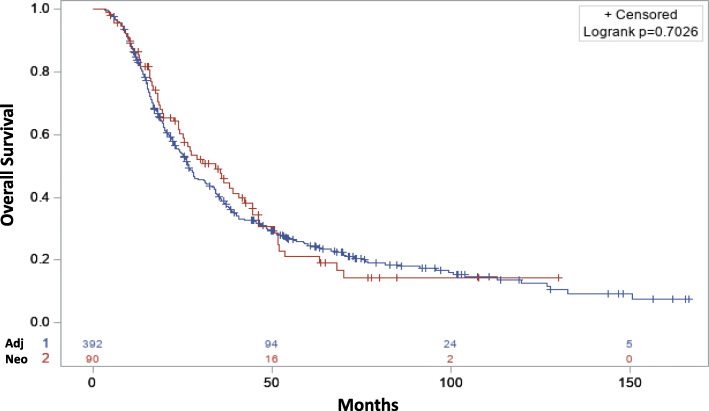
Overall survival neoadjuvant (red) vs. adjuvant (blue) immunotherapy for the entire cohort

The KM curves based on the log-rank test did not show any significant difference in the median OS of patients who received neoadjuvant immunotherapy compared to adjuvant immunotherapy (Fig. 1). The median OS of patients who received neoadjuvant immunotherapy was 26.78 months (CI: 23.92–31.24) vs. 34.37 months (CI: 24.21–42.28 months; *p* = 0.703) in patients who received adjuvant immunotherapy. In the multivariable Cox analysis, neoadjuvant immunotherapy was not associated with improved OS (HR: 1.06, CI: 0.79–1.41; *p* = 0.714) compared to adjuvant immunotherapy (Table 2).

**Table 2:** Univariate and multivariate Cox regression analysis of neoadjuvant immunotherapy vs. adjuvant immunotherapy

Variable		N (%)	Univariable analysis		Multivariable analysis	
			Hazard Ratio (95% CI)	P	Hazard Ratio (95% CI)	P
Immunotherapy	Neoadjuvant immunotherapy	408 (77.57)	1.06 (0.80–1.39)	0.703	1.06 (0.79–1.41)	0.714
	Adjuvant immunotherapy	118 (22.43)	Ref		Ref	

The multivariable analysis was adjusted for the age at diagnosis, sex, race, income, education, place of living, treatment facility type, insurance status, comorbidity score, and year of diagnosis

### Subset analyses

#### Neoadjuvant subset analysis

**Fig. 2:**
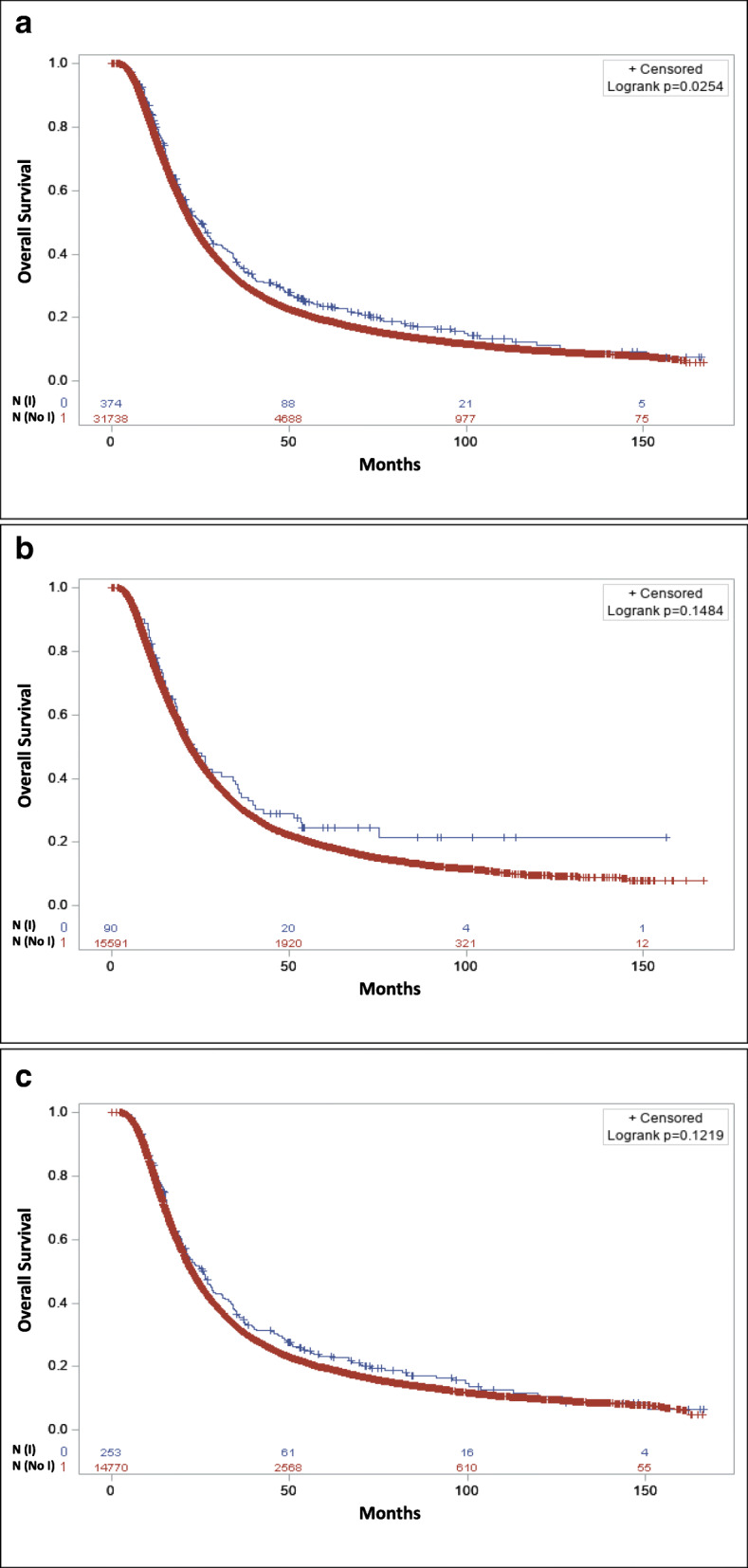
Overall survival with (blue) or without (red) neoadjuvant immunotherapy for (**a**) all patients with only neoadjuvant therapies; **b** patients who received neoadjuvant chemotherapy; **c** patients who received neoadjuvant chemoradiation

This group was restricted to patients who only received neoadjuvant treatments such as chemotherapy, RT, chemoradiation, and immunotherapy. If any of the treatment was not neoadjuvant, those observations were excluded from this subset analysis. Based on the log-rank analysis, patients who received neoadjuvant immunotherapy had significantly improved OS with an absolute median OS benefit of 2.6 months compared to patients who did not receive immunotherapy (25.10 months, CI: 21.42–27.96 vs. 22.51 months, CI: 22.21–22.77; *p* < 0.025) (Fig. 2a). In the univariate Cox proportional analysis neoadjuvant immunotherapy was associated with improved OS (HR: 0.88, CI: 0.78–0.98; *p* < 0.026) compared to no immunotherapy. However, in the multivariable analysis, this association became nonsignificant (Table 3). In order to find the explanatory factor in the multivariate analysis, which was responsible for the loss of significance in the effect of immunotherapy on OS, we performed stepwise regression analysis to determine the factor(s) which may interact with neoadjuvant immunotherapy. In the stepwise regression analysis, age at diagnosis, income, education, and hospital type showed interaction with immunotherapy, and the effect of immunotherapy on OS became insignificant when any of these variables were added to the model with immunotherapy. We then stratified by the categories of each of these variables and found that immunotherapy only stayed significant in patients who were living in areas with < 13% people with no high school degree. After patients were stratified by educational level, we found that in patients who live in areas with a high level of education, the impact of immunotherapy on OS stayed significant despite income, age at diagnosis, and hospital type. In the multivariable Cox regression analysis, neoadjuvant immunotherapy was associated with significantly improved OS (HR: 0.86, CI: 0.74–0.99; *p* < 0.04) compared to no immunotherapy only in patients with high-level of education, but not in patients with low-level of education.

**Table 3:** Cox regression analysis of only neoadjuvant immunotherapy combinations

Variable		N (%)	Univariable analysis		Multivariable analysis	
			Hazard Ratio (95% CI)	P	Hazard Ratio (95% CI)	P
Immunotherapy	Neoadjuvant immunotherapy	373 (1.09)	0.88 (0.78–0.98)	0.026	0.93 (0.82–1.05)	0.220
	No immunotherapy	33,921 (98.91)	Ref		Ref	
CTx plus immunotherapy	Neoadjuvant CTx plus imm	95 (0.53)	0.835 (0.653–1.067)	0.150	0.930 (0.725–1.195)	0.572
	Adjuvant CTx only	17,868 (99.47)	Ref			
CTxRT plus immunotherapy	Neoadjuvant CTxRTx plus imm	258 (1.64)	0.897 (0.781–1.030)	0.122	0.942 (0.814–1.091)	0.425
	Adjuvant CTxRTx only	15,466 (98.36)	Ref			

*CTx:* chemotherapy, *CTxRTx:* chemoradiation therapy, *imm:* immunotherapy

Further analysis revealed that there was no difference in the median OS of patients who received neoadjuvant chemotherapy plus immunotherapy compared to neoadjuvant chemotherapy alone (Fig. 2b) and patients who received neoadjuvant chemoradiation plus immunotherapy compared patients who received only neoadjuvant chemoradiation (Fig. 2c). In the multivariable analysis, there was no difference in the OS of patients who received neoadjuvant chemotherapy plus immunotherapy compared to neoadjuvant chemotherapy alone (HR: 0.930, CI: 0.725–1.195; *p* = 0.972) and patients who received neoadjuvant chemoradiation plus immunotherapy compared to neoadjuvant chemoradiation alone (HR: 0.942, CI: 0.814–1.091; *p* = 0.425) (Table 3).

#### Adjuvant subset analysis

**Fig. 3:**
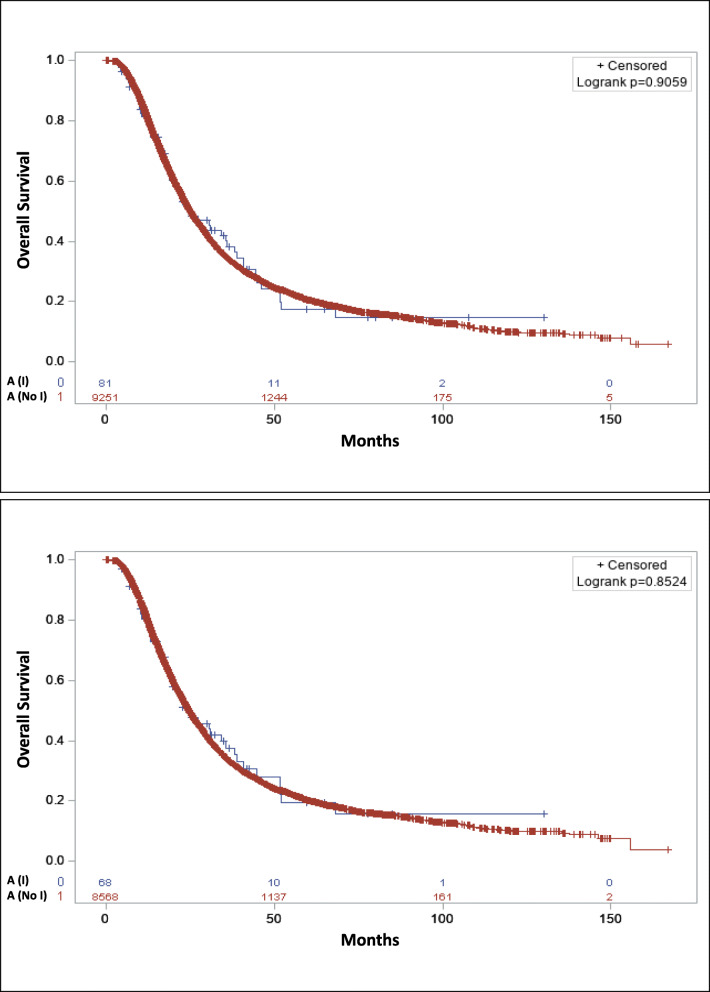
Overall survival with (blue) or without (red) adjuvant immunotherapy for (**a**) all patients with only adjuvant therapies; **b** patients who received adjuvant chemotherapy or adjuvant chemoradiation

This analysis included patients who only received adjuvant chemotherapy, RT, chemoradiation, and immunotherapy. If any of the treatment was not adjuvant those patients were not included in this subset analysis. Based on KM curves there was no difference in the median OS of patients who received adjuvant immunotherapy compared to patients who received other adjuvant treatment but did not receive immunotherapy (Fig. 3a). There was no difference in the median OS of patients received adjuvant chemotherapy plus immunotherapy or chemoradiation plus immunotherapy compared chemotherapy or chemoradiation without immunotherapy (Fig. 3b). In the multivariable analysis, there was no significant difference in the OS of patients who received adjuvant immunotherapy compared to no immunotherapy (HR:1.00, CI: 0.76–1.32; *p* = 0.995). A significant difference in the OS was also not observed between patients who received adjuvant chemotherapy plus immunotherapy or chemoradiation plus immunotherapy compared chemotherapy or chemoradiation without immunotherapy (HR: 1.01, CI: 0.75–1.37; *p* = 0.935) (Table 4). The adjuvant chemotherapy plus immunotherapy group was combined with chemoradiation plus immunotherapy due to a small sample size.

**Table 4:** Cox regression analysis of only adjuvant immunotherapy combinations

Variable		N (%)	Univariable analysis		Multivariable analysis	
			Hazard Ratio (95% CI)	P	Hazard Ratio (95% CI)	P
Immunotherapy	Adjuvant immunotherapy	106 (0.96)	0.98 (0.76–1.28)	0.907	1.00 (0.76–1.32)	0.995
	No immunotherapy	10,950 (99.04)	Ref		Ref	
CTx or CTxRTx plus immunotherapy	Adjuvant CTx or CTxRTx plus imm	90 (0.88)	0.97 (0.73–1.30)	0.854	1.01 (0.75–1.37)	0.935
	Adjuvant CTx or CTxRTx	10,104 (99.12)	Ref		Ref	

We combined adjuvant chemotherapy plus immunotherapy with adjuvant chemoradiation plus immunotherapy due to small sample size. When analyzed separately the results were the same. CTx: chemotherapy, CTxRTx: chemoradiation therapy, imm: immunotherapy

## Discussion

To our knowledge, the current study is the most extensive study that has compared the impact of neoadjuvant immunotherapy vs. adjuvant immunotherapy on the OS of PDAC patients who received definitive surgery of the pancreatic tumor. There was no significant difference in the median OS of patients who received neoadjuvant immunotherapy compared to patients who received adjuvant immunotherapy. However, in the neoadjuvant subset analysis, immunotherapy was associated with significantly improved OS compared to no immunotherapy in the univariate analysis though this significance was lost upon multivariable analysis.

The improved OS of neoadjuvant immunotherapy compared to no immunotherapy in the univariate analysis may be due to the impact of immunotherapy in eradicating the occult micrometastases that occur early in PDAC [1, 2]. The tumor cells use mechanisms such as the up-regulation of immune checkpoint signaling programmed death-ligand 1 (PD-L1), downregulation of cytotoxic T-lymphocyte-associated protein 4 (CTLA4), and the recruitment of MDSCs, to evade the immune system [43–45]. Immunotherapy, especially checkpoint inhibitors, down-regulates the PD-L1 pathway and upregulates anti-CTLA4 [16, 19]. The insignificant results of neoadjuvant immunotherapy compared to adjuvant immunotherapy may indicate that the impact of immunotherapy on the OS of PDAC patients who receive definitive surgery of the pancreatic tumor is not related to the sequence of immunotherapy with surgery. A small sample size of group comparisons in the neoadjuvant and adjuvant subsets analyses may be responsible for insignificant results. Neoadjuvant chemotherapy plus immunotherapy was not associated with improved OS compared to neoadjuvant chemotherapy. However, the extended plateaued or nearly a flat line at the end of the KM curve is indicative of the long-lasting immunity or cure from cancer, which is only seen in patients who received both chemotherapy and immunotherapy (Fig. 2b, blue). This is strong evidence of long-lasting immunity and possible cure from cancer in patients who received neoadjuvant chemotherapy plus immunotherapy.

The year of diagnosis could interact with both chemotherapy and immunotherapy. Treatment with chemotherapy and immunotherapy has evolved over the years and will likely influence the OS of the resectable pancreatic cancer patients. The 5-year survival rate after resection improved to 16–21% in pancreatic patients who receive adjuvant chemotherapy with gemcitabine or 5-fluorouracil plus folinic acid after resection compared to 10% for surgery alone [8]. Median survival time of up to 54 months has been reported with adjuvant modified FOLFIRINOX in resected pancreatic cancer patients [4]. Checkpoint inhibitors, the most widely used immunotherapy, became available only after 2013. Patients who were diagnosed after 2013 are likely to have received a checkpoint inhibitor and may have better OS compared to patients who either received other types of immunotherapy or were diagnosed before 2013. We performed separate multivariable analyses for patients who were diagnosed after 2013 and patients who were diagnosed before 2013. In both stratified analyses, there was no difference in the OS of patients who received neoadjuvant immunotherapy and patients who received adjuvant immunotherapy. There was not enough sample size to stratify by individual year.

### Limitations

The large sample size for the Comparison of neoadjuvant immunotherapy vs. adjuvant immunotherapy is the most important strength of the current study, which allowed us to adjust for patient and tumor characteristics. However, the study is not without limitations, most of which are inherent to NCDB and include selections bias, lack of information of the cause of death, lack of information about the type of immunotherapy and if a single or combined immunotherapy was administered, and lack of detailed information on the use of multi-agent chemotherapy. Another limitation is the inability to calculate progression-free survival as the NCDB does not provide information on disease progression or recurrence. One other limitation was that due to the small sample size for immunotherapy plus RT, the sequence of immunotherapy with RT alone was not performed. In addition, there were not enough cases for adjuvant comparison, and that maybe one of the reasons that we failed to find any significant difference in the OS of patients who received adjuvant immunotherapy, chemotherapy plus immunotherapy, and chemoradiation plus immunotherapy compared to their counterparts without immunotherapy.

Nonetheless, in this study, a robust analysis of the impact of the timing of immunotherapy with surgery on the OS of PDAC patients who received definitive surgery of the pancreatic tumor using the NCDB was performed. The NCDB is the largest cancer database in the world which captures the majority of the annual cancer cases diagnosed in the U.S. It serves as an outstanding source for the investigation of the impact of novel cancer treatments on the OS of cancer patients.

## Conclusions

No difference in the OS between patients who received neoadjuvant immunotherapy and those who receive adjuvant immunotherapy was noticed. However, in the univariate analysis, neoadjuvant immunotherapy was associated with significantly improved OS compared to no immunotherapy. The findings warrant future studies with a large sample size for both neoadjuvant and adjuvant treatment comparisons of immunotherapy.

## Data Availability

The datasets used and analyzed during the current study are available from the corresponding author on reasonable request.

## Abbreviations

NCDB: National Cancer Database
OS: Overall survival
PDAC: Pancreatic adenocarcinoma
MDSCs: Myeloid-derived suppressor cells
RT: Radiation therapy
PD-L1: Programmed death-ligand 1
CTLA4: Cytotoxic T-lymphocyte-associated protein 4
CTx: Chemotherapy
Imm: Immunotherapy
CTxRTx: Chemoradiation therapy
RTx: Radiation therapy
